# Novel loss-of-function variants in *WDR26* cause Skraban-Deardorff syndrome in two Chinese patients

**DOI:** 10.3389/fped.2024.1429586

**Published:** 2024-09-18

**Authors:** Qi Yang, Xunzhao Zhou, Sheng Yi, XiaoLing Li, Qiang Zhang, Shujie Zhang, Li Lin, Shang Yi, Biyan Chen, Zailong Qin, Jingsi Luo

**Affiliations:** ^1^ Guangxi Key Laboratory of Birth Defects Research and Prevention, Guangxi Key Laboratory of Reproductive Health and Birth Defects Prevention, Maternal and Child Health Hospital of Guangxi Zhuang Autonomous Region, Nanning, China; ^2^ Department of Genetic and Metabolic Central Laboratory, Maternal and Child Health Hospital of Guangxi Zhuang Autonomous Region, Nanning, China; ^3^ Department of Child Health Care, Maternal and Child Health Hospital of Guangxi Zhuang Autonomous Region, Nanning, China; ^4^ Guangxi Clinical Research Center for Pediatric Diseases, Maternal and Child Health Hospital of Guangxi Zhuang Autonomous Region, Nanning, China

**Keywords:** novel *de novo* variants, *WDR26*, Skraban-Deardorff syndrome, intellectual disability, developmental delay, hypotonia, behavioural problems, seizures

## Abstract

**Introduction:**

Mutations in the protein WD repeat structural domain 26 (*WDR26*, MIM 617424) have been identified as the cause of autosomal dominant Skraban-Deardorff syndrome, a rare genetic disorder characterized by intellectual disability (ID), developmental delay (DD), hypotonia, epilepsy, infant feeding difficulties, gait abnormalities and distinctive facial features. The objective of this study is to investigate the genetic factors that may contribute to the development of Skraban-Deardorff syndrome in affected individuals.

**Methods:**

In this study, we used whole-exome sequencing (WES) to analyze pathogenic and likely pathogenic variants in two unrelated Chinese patients with DD and ID. We confirmed the origin of the variants by conducting Sanger sequencing and classified them according to ACMG/AMP guidelines.

**Results:**

Here, two novel *de novo* variants (c.1797delC(p.His599fs*11) and c.1414C>T(p.Gln472*)) in the *WDR26* gene have been identified in two Chinese patients with Skraban-Deardorff syndrome. These patients exhibit a range of symptoms, including varying degrees of ID, DD, speech delay, an abnormal wide-foot and/or stiff-legged gait, facial dysmorphism, behavioural abnormalities, with or without seizures.

**Conclusions:**

In this study, We report two unrelated Chinese patients with Skraban-Deardorff syndrome caused by novel *de novo* pathogenic variants of the *WDR26* gene. These patients showed a clinical phenotype similar to that of patients with the *WDR26* variant. Compared to reported cases with *WDR26* pathogenic variants, patient 2 presented a novel complication of severe behavioural problems, including hyperactivity, social anxiety, self-mutilation, impulsivity and violent behaviour. This research broadens the range of genetic and clinical features of Skraban-Deardorff syndrome. In addition, the symptoms may become more pronounced as the patient ages. Furthermore, our report highlights the clinical diversity of Skraban-Deardorff syndrome. The findings may assist healthcare professionals in providing more accurate genetic testing and counselling to affected families and improving the overall management of the condition.

## Introduction

**Figure 1:**
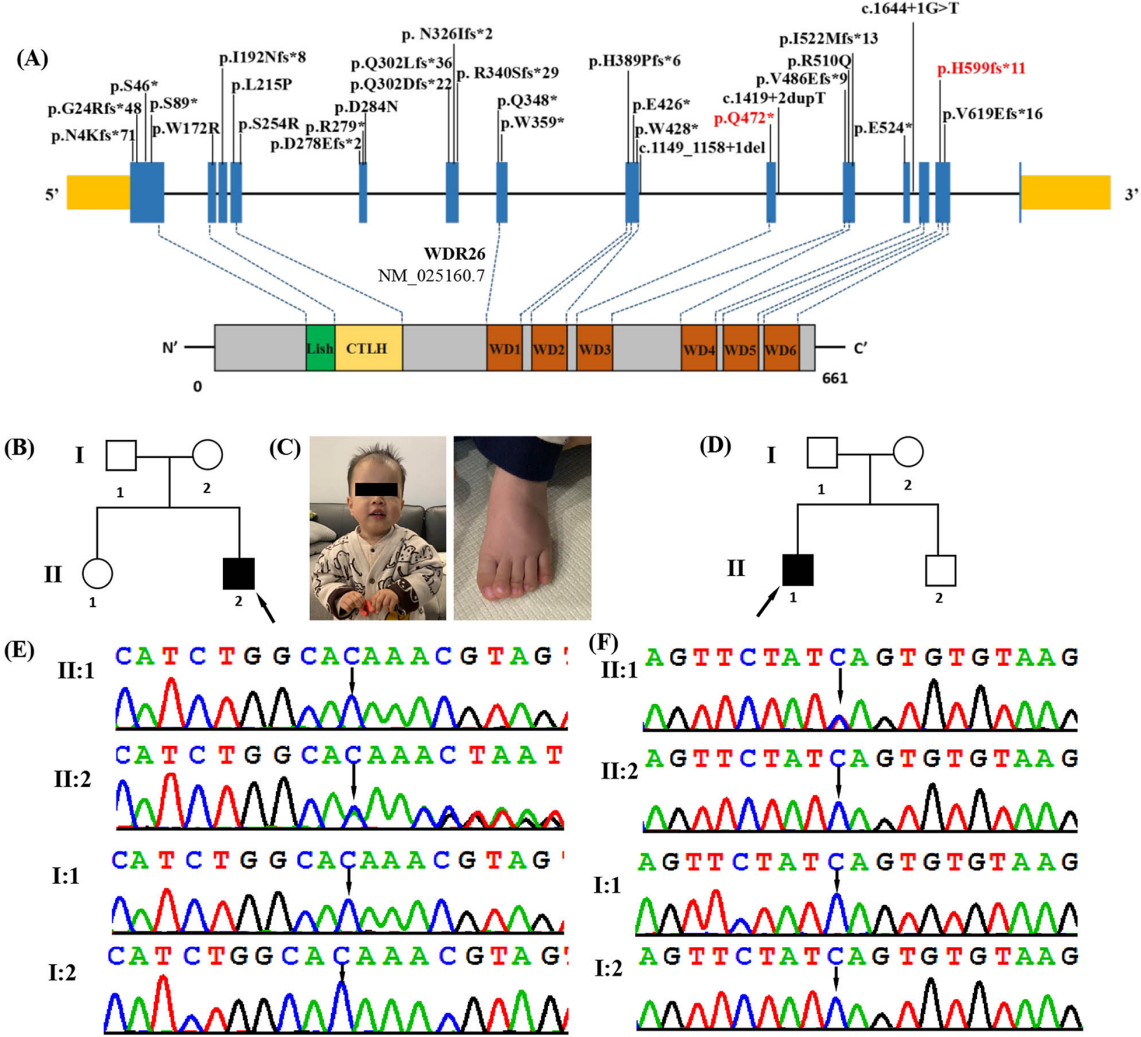
Clinical and genetic features. **(A)** The distribution of all *WDR26* variants detected so far in the 26 reported patients and 2 in this study. Boxes represent 14 different exons as indicated, and solid lines connecting these boxes represent the introns of *WDR26* gene (NM_025160.7). The variants in our patients are highlighted in red. Schematic diagram of the location of the *WDR26* gene domains. **(B)** Pedigree of the affected family 1. Arrow indicates the proband (II2). **(C)** Photograph of patient 1 (family 1, II-2) at the age of 1 year 6 months showing frontal bossing, full cheeks, downslanting palpebral fissures, coarse facial features, depressed nasal root, full nasal tip, long philtrum and tented upper lip vermilion. Dysmorphic features also included left curved hallux phalanx. **(D)** Pedigree of the affected family 2. Arrow indicates the proband (II1). **(E)** DNA sequence chromatograms obtained through Sanger sequencing of *WDR26* revealed a *de novo* heterozygous variant c.1797delC(p.His599fs*11) in patient 1(I:2), which was not detected in either his parents (II:1 and II:2) or his sister (I:1). **(F)** DNA sequence chromatograms from Sanger sequencing of *WDR26*, showing a *de novo* heterozygous variant c.1414C>T(p.Gln472*) in patient2 (I:1). Further Sanger sequencing indicated that the variant was not identified in his parents (II:1 and II:2) and brother (I:2).

Skraban-Deardorff syndrome (MIM #617616) is a rare genetic disorder that is inherited in an autosomal dominant manner (1). It is characterized by a variety of symptoms including intellectual disability, developmental delay, hypotonia, epilepsy, infant feeding difficulties, abnormal gait, and distinct facial features such as a depressed nasal root, broad nasal tip, anteverted nares, prominent smile, widely spaced teeth, and gingival abnormalities (1–4). The disorder is caused by mutations in the WD Repeat Domain 26 (*WDR26*, MIM 617424) gene, located at 1q42.12 (1). This gene contains 14 exons and encodes a WD-repeat protein with six WD repeat motifs (Figure 1A). *WDR26* is highly conserved in mammalian cells and plays a crucial role in regulating various cellular processes, such as MAPK, Wnt and PI3 K signaling, neuronal and cardiomyoblast proliferation, apoptotic signaling, as well as leukocyte activation and signal transduction (5–9).

To date, only 28 loss-of-function (LoF) *WDR26* mutations have been identified in patients with Skraban-Deardorff syndrome (Figure 1A) (1–4, 10–12). These mutations comprise five missense variants, twelve frameshift variants, eight nonsense variants, and three splicing variants. The mutations in *WDR26* can result in a wide range of phenotypic defects that can vary from neurological manifestations to non-neurological ones. These defects may include seizures, developmental delay, intellectual disability, short stature, speech delay, motor delay, gait abnormalities, characteristic facial features, and multiple structural anomalies. Due to the limited number of previously reported patients and associated clinical information, there is a need for more reports on WDR26 mutations and their phenotypes, which could contribute to a better understanding the disease. In this study, we describe two additional cases of patients with Skraban-Deardorff syndrome who had two *de novo* heterozygous mutations in the *WDR26* gene (NM_025160.7; c.1797delC(p.His599fs*11), c.1414C>T(p.Gln472*)). We also provide additional molecular and clinical information to enhance our understanding of Skraban-Deardorff syndrome.

## Materials and methods

### Ethical compliance

The study was carried out with utmost adherence to the principles of the Declaration of Helsinki, and received approval from the esteemed Institutional Review Board and Ethics Committee of Guangxi Maternal and Child Health Hospital (GXMC20230608). The patients’ families provided written informed consent.

### Whole-exome sequencing and sanger sequencing

Peripheral blood samples were taken from the patients and their family members and the genomic DNA was extracted from the samples using the Lab-Aid DNA Kit (Zeesan Biotech Co., Ltd., Xiamen, China). Whole exome enrichment was then performed using Agilent SureSelect V5 (Agilent Technologies, Santa Clara, CA, USA). The enrichment libraries underwent sequencing using the Hiseq2000 platform and generated 100-bp paired-end reads. The sequence reads obtained were aligned to the human genome assembly GRCh38 by using the Burrows-Wheeler Aligner (BWA-MEM, version 0.7.10). Variant calling was performed using the Genome Analysis Toolkit 3.4 (GATK), and all the variants were annotated and classified using TGEX. Filtering criteria were employed to select the variants based on: (i) variants with a frequency below 0.5% in public databases such as Exome Sequencing Project, the 1,000 Genomes Project, ExAC, and in-house databases, (ii) exonic variants with non-synonymous changes, and intronic variants in 10-bp exon flanking regions, and (iii) potentially deleterious variants predicted by functional prediction tools such as PROVEAN, PolyPhen2, CADD, and MutationTaster. The candidate variants obtained from the whole-exome sequencing were validated by Sanger sequencing in both the patients and their parents. The identified variants were scored and classified according to the guidelines of the American College of Medical Genetics and Genomics (ACMG) and the Association for Molecular Pathology (AMP) (13).

## Results

### Clinical features

Patient 1, a male aged 1 year 6 months, was the second child of healthy, non-consanguineous Chinese parents (Figure 1B, Table 1). He was born at the normal gestational age (39 weeks) via induced vaginal delivery with a normal birth weight (3.6 kg) and birth length (48.9 cm). His development was delayed as he started sitting at 9 months, crawling at 10 months, and did not walking without support until 18 months. He began to speak a few words at 1-year-5-months-old, such as “baba” and “mama”. The patient was found to have a mild global developmental delay. The Gesell Developmental Scale for Children was used at 1 years of age and the patient's developmental quotients (DQs) in the domains of adaptation, gross motor, fine motor, language and social behaviour were 65, 62, 60, 62 and 85, respectively. On physical examination, he had a length of 86.2 cm (>1SD), a weight of 12 kg, and a head circumference of 49.1 cm (>2SD). His personality was described as friendly, without autism or seizures. Facial dysmorphic features were observed and included frontal bossing, full cheeks, downslanting palpebral fissures, coarse facial features, depressed nasal root, full nasal tip, long philtrum and tented upper lip vermilion (Figure 1C). Dysmorphic features also included decreased palmar creases and a curved left hallux phalanx (Figure 1C). He also had hypotonia. His brain MRI and chest x-ray were normal. His G-band karyotype was determined to be 46, XY.

**Table 1:** Clinical features of the patient with *de novo WDR26* mutations.

Clinical features	Patient 1	Patient 2
Variants in *WDR26* (NM_025160.7)	c.1797delC(p.His599fs*11)	c.1414C>T(p.Gln472*)
Gender	Male	Male
Age at last examination	1 years and 6 months	11 years
Gestation	Full-term	Full-term
Muscular hypotonia	Yes	Yes
Weight	12 kg (ref:10.65 kg)	29 kg (<−1SD;36.10 kg)
Height	86.2 cm (>1SD; ref: 81.5 cm)	138 cm (<−1SD; ref:146.6 cm)
Head circumference	49.1 cm(>2SD;ref:47.8 cm)	NA
Developmental delay/movement delay	Yes; mild	Yes; severe
Intellectual disability	Yes; mild	Yes; profound
Hypotonia	Yes	Yes
Age of walking	18 months	25 months
Age of first words	17 months	36 months and currently has no meaningful language
Behavior anomalies	Friendly, without autism	Cognitive impairment, hyperactivity, social anxiety, self-mutilation, impulsive, violent and autistic-like behaviours
Facial dysmorphisms	Frontal bossing, full cheeks, downslanting palpebral fissures, coarse facial features, depressed nasal root, full nasal tip, long philtrum and tented upper lip vermilion	Mildly coarse facial features with full cheeks, sparse lateral brows, open mouth posture, mild micrognathia, widely spaced teeth and tented upper lip vermilion
Electroencephalogram	Normal	Intermittent discrete spike/sharp wave discharges over the right occipital and left middle temporal regions
Seizures	No	Yes (tonic-clonic seizures)
Other anomalies	Decreased palmar creases and left curved hallux phalanx	Pes planus

Patient 2 was a male who was first seen at the Department of Paediatrics, Maternal and Child Health Hospital of Guangxi Zhuang Autonomous Region at the age of 11 years for genetic counselling regarding ID and DD (Figure 1D, Table 1). Pitt Hopkins Syndrome, α-Thalassaemia X-linked Intellectual Disability Syndrome and Mowat Wilson Syndrome had been suspected in this patient when he was treated for ID and DD rehabilitation in other hospitals, but no genetic testing had been performed. He was the first of two children and there was no no family history of ID or developmental delay. The patient was born at 38 weeks gestation, with a normal birth weight (3,200 g). His development was globally delayed. He started to roll over at 8 months, sit unsupported at 9 months, crawl at 11 months and walk at 2 years and 1 months, but still has a stiff-legged gait. His speech was also significantly delayed, speaking single words by the age of 3 and currently has no meaningful language. He had his first seizure at the age of 2. He developed tonic-clonic seizures at the age of two and was treated with sodium valproate (VPA) and levetiracetam (LEV) without further seizures. The electroencephalogram (EEG) showed intermittent discrete spike/sharp wave discharges over the right occipital and left mildly temporal regions. Brain MRI was normal. Physical examination revealed mildly coarse facial features with full cheeks, sparse lateral brows, open mouth posture, mild micrognathia, widely spaced teeth, tented upper lip vermilion, pes planus and hypotonia. He had a severe intellectual disability. According to the Wechsler Intelligence Scale for Children at the age of 11 years, his Full Scale IQ was 26. He exhibited some abnormal behaviours including cognitive impairment, hyperactivity, social anxiety, self-mutilation, impulsive, violent and autistic-like behaviours.

### Molecular analysis

To determine the genetic cause of the disease, we performed whole exome sequencing on these patients. A total of 14.7 Gb of data was generated, with 99.5% coverage of the target region and 99.0% of targets covered more than 20×. After retaining single nucleotide variations (SNVs) or insertions/deletions (indels) in coding regions and splice sites (10 bp from the splice junctions), and filtering out synonymous variations and variations with an allele frequency (MAF) greater than 0. 1% in public databases (such as the 1,000 Genomes Project, Genome Aggregation Database, Exome Sequencing Project and ExAC) and our internal database, we used the TGex software (LifeMap Sciences, USA) to map 11 candidate variations in 10 genes (*MBD5*, *LAMA1*, *CDON*, *PLXNA1*, *ASH1l*, *GABBR2*, *TBR1*, *CHAMP1*, *JAG2* and *WDR26*) to the known phenotypes (including global developmental delay, delayed speech and language development, autistic behaviour, behavioural abnormalities, hypotonia, seizures and intellectual disability), and then extracted the variations (Supplementary Table S1). As a result, a heterozygous frameshift variant located in exon 13 of *WDR26* (NM_025160.7: c.1797delC/p.His599fs*11)) was identified in patient 1 (Figure 1E). A heterozygous nonsense variant located in exon 9 of *WDR26* [c.1414C>T(p.Gln472*)] was identified in patient 2 (Figure 1F). We validated the two variants by Sanger sequencing. We also sequenced the parental samples to identify the variants as *de novo*. These variants were also not reported in any of the following databases: Human Gene Mutation Database, Exome Sequencing Project, gnomAD, ClinVar, 1,000 Genomes Project, and Single Nucleotide Polymorphism Database. Both variants were predicted to be deleterious using in silico tools. Table 2 shows the pathogenicity prediction analysis and ACMG/AMP score of the two WDR26 variants.

**Table 2:** Predicted pathogenicity of *de novo WDR26* variants.

Patient	Variant (NM_025160.7)	Inheritance	LRT	Mutationtaster	NMDEscPredictor	PolyPhen-2	SIFT	CADD	ACMG/AMP
Patient 1	c.1797delC(p.His599fs*11)	DNV	N.A.	D	NMD	N.A.	N.A.	N.A.	P(PVS1 + PS2 + PM2)
Patient 2	c.1414C>T(p.Gln472*)	DNV	D	D	NMD	N.A.	N.A.	N.A.	P(PVS1 + PS2 + PM2)

DNV, *de novo* variant; D, deleterious or damaging; NMD, nonsense mediated decay; PD, probably damaging; N.A. not available; P, pathogenic.

## Discussion

Skraban-Deardorff syndrome is a rare inherited neurodevelopmental disorder. In 2017, Skraban et al. first reported the identification of *WDR26* variants in 15 patients with ID and DD using WES (1). Subsequently, Pavinato et al. further expanded the clinical phenotype of the ultra-rare Skraban-Deardorff syndrome by investigating *WDR26* variants in two patients with neurodevelopmental disorder and other multiple malformations (2). To date, only 28 individuals with Skraban-Deardorff syndrome have been reported (1–4, 10–12). In this study, two unrelated Chinese patients were diagnosed with Skraban-Deardorff syndrome after whole-exome sequencing (WES) analysis revealed two novel *de novo WDR26* gene variants. Both patients had similar symptoms, including ID, DD, delayed speech, wide-base and/or stiff-legged gait, facial dysmorphism. Therefore, the patients were diagnosed with Skraban-Deardorff syndrome.

The clinical features of 30 patients with Skraban-Deardorff syndrome, including the patients in this study, are summarised in Table 3. Some common clinical manifestations were observed. All the patients exhibited intellectual disability and/or developmental delay, ranging from mild to severe. 74.1% (20/27) of the patients presented with hypotonia, and all of them learned to walk, albeit some with gait abnormalities. Speech delay is likely the most severely impacted aspect, with all the patients experiencing speech delay and one patient remaining non-verbal at the age of 8 years (1). All patients possessed identifiable dysmorphic facial features that seemed to become more characteristic with age. These features included coarse facial features (21/29, 72.4%), large irises or rounded/short/slanting palpebral fissures (18/29, 62.1%), abnormal eyebrows (14/29, 48.3%), depressed nasal root (16/29, 55.2%), anteverted nasal bridge (17/29, 58.6%), broad/full nasal tip (23/29, 79.3%), protruding upper lip (19/29, 65.5%), protruding or full/tense upper lip (20/29, 69%), reduced cupid's bow (19/29, 65.5%), widely spaced teeth (23/29, 79.3%) and abnormal gums (18/29, 62.1%). Most of the patients had feeding problems in infancy, which improved later. Seizures, with or without EEG abnormalities, were present in 80% (24/30) of the patients. The age of onset of epilepsy ranged from the neonatal period to 7 years. Five of the patients (including one of our patients) were seizure-free. This suggests that the presence of epilepsy in patients with Skraban-Deardorff syndrome may not be universal. Behavioural abnormalities were also common, with 65.2% (15/23) of patients exhibiting autism or repetitive, posturing, or rocking behaviour. Brain abnormalities included a variety of central nervous system (CNS) malformations, ranging from pineal cysts and arachnoid cysts to periventricular white matter softening, observed in 65.5% (19/29) of patients. Skeletal deformities were also a common feature of the disease (51.7%, 15/29), and a wide spectrum of skeletal anomalies were observed, including clinodactyly, pes planus, and scoliosis.

**Table 3:** Main clinical features in our patients and comparison with the 23 reported WDR-26 patients in literatures.

Feature	Our patient		Skraben et al., 2017	Pavinato et al., 2021	Cospain et al., 2021	Chen et al., 2022	Innella et al., 2022	Hu et al., 2022	Gunasekaran et al., 2024	Total
Patient	Patient 1	Patient 2	15 patients	2 patients	6 patients	1 patient	1 patient	2 patients	1 patient	30
Gender (male:female)	Male	Male	5 male:10 female	2 female	3 male:3 female	1 male	1 female	1 male:1 female	1 male	13 male: 17 Female
Age at presentation/diagnosis	1 year 6 months	11 years	24months-34years	8years and 19years	18months-16years	18months-34years	18 years	11 years and 3 years	8 years	
Developmental delay/intellectual disability	+	+	15/15	2/2	6/6	+	+	2/2	+	30/30
Epilepsy/EEG abnormalities	-	+	15/15	2/2	5/6	-	-	0/2	+	24/30
CNS structural anomalies	-	-	10/14	1/2	4/6	-	+	2/2	+	19/29
Hypotonia	+	+	9/12	1/2	4/6	+	-	2/2	+	20/27
Abnormal gait	+	+	9/15	2/2	3/6	+	-	2/2	NA	19/29
Limited speech	+	+	15/15	2/2	6/6	+	+	2/2	+	30/30
Happy and/or friendly personality	+	-	10/15	2/2	6/6	+	-	2/2	NA	22/29
Autistic and/or repetitive behaviors or posturing	+	+	5/9	1/2	5/6	-	+	1/2	NA	15/23
Coarse facial features	+	+	12/15	1/2	2/6	+	+	2/2	NA	21/29
Full cheeks as a child	-	+	11/13	2/2	5/6	+	+	1/2	NA	22/27
Large irises or rounded/short/slanting palpebral fissures	+	-	10/15	1/2	4/6	-	-	2/2	NA	18/29
Abnormal eyebrows	-	+	6/15	1/2	4/6	+	+	0/2	NA	14/29
Depressed nasal root	+	-	5/15	2/2	5/6	+	-	2/2	NA	16/29
Anteverted nares	+	-	8/15	2/2	5/6	-	-	1/2	NA	17/29
Broad/Full nasal tip	+	+	11/15	2/2	6/6	-	+	2/2	NA	23/29
Prominent maxilla and protruding upper lip	-	+	13/15	0/2	3/6	+	+	0/2	NA	19/29
protruding or full, tented upper lip	+	+	13/15	0/2	3/6	+	+	0/2	NA	20/29
Decreased cupid's bow	+	-	11/15	1/2	5/6	-	-	1/2	NA	19/29
Widely spaced teeth	+	+	13/15	2/2	3/4	+	+	1/2	NA	23/29
Abnormal gums	+	-	9/15	2/2	2/2	+	+	2/2	NA	18/29
Ophthalmologic abnormalities	-	-	9/14	NA	1/6	+	+	0/2	+	13/28
Nail hypoplasia	-	-	3/13	NA	NA	NA	NA	0/2	NA	3/18
Short stature	-	+	2/15	0/2	1/6	-	+	0/2	+	6/30
Digit abnormalities	+	-	3/15	1/2	1/6	-	-	0/2	NA	6/29
Gastrointestinal dysfunctions	+	-	5/15	0/2	NA	NA	NA	0/2	NA	6/21
Orthopaedic disorders	+	+	5/15	2/2	4/6	+	+	0/2	NA	15/29

CNS, central nervous system; EEG, electroencephalogram; NA: not applicable.

Although we observed convergent phenotypes in our patients and in previous cases, we also noted that patients carrying the *WDR26* variant can present with diverse phenotypes even when harboring the same type of variant. In the present study, both patient 1 and patient 2 carry the null mutation, and patient 2 exhibits more significant developmental delay, severe speech delay, profound intellectual disability, seizures and behavioural abnormalities. However, patient 1 had a milder phenotype, including mild mental retardation, motor and speech delays, and no behavioural problems or seizures. In addition, the patient carrying the variant (p.V619Efs*16), located in the same exon as patient 1, displays more severe clinical manifestations (3). The phenotypic heterogeneity in previous cases were also observed by Skraban et al. (1). The diverse phenotypes could be caused by individual differences in the patients or by the positional effects of the variants.

Interestingly, we observed relatively milder symptoms such as intellectual disability, developmental delays, and mild facial deformities in all six children (including patient 1) who were 4 years of age or younger (1, 3, 4). In contrast, a more severe phenotype was noted in some older patients. This seemingly indicate that the patient's presentation tends to becomes more pronounced with age. However, the scarcity of reports on patients with long-term phenotypes, we are currently unable to demonstrate the relationship between the phenotype and the age of the patient. It is anticipated that the phenotype will be further elucidated as the number of reported patients continues to rise and the results of long-term patient follow-up are made available, offering a more lucid depiction of the influence of genotype and other determinants on the phenotype.

Early-onset epilepsy, with an age of onset ranging from infancy to 7 years of age, has been observed in almost all patients with Skraban-Deardorff syndrome (1–3, 9). Various types of seizures have been observed, with generalised motor seizures and non-motor absences seizures being the most common (1–3, 9). In this study, patient 2 presented with Generalized Tonic-Clonic Seizure (GTCS) at the age of two years. However, no seizures were observed in patient 1 at the time of the last examination. Considering that the patient was only 1 year and 6 months old, there needs to be concern that the patient may develop epilepsy with age. Although seizures often correlate with the severity of intellectual disability, patient 2 did not have any improvement in cognitive or developmental outcomes after his seizures were under control. Notably, some individuals without seizures still exhibit moderate to severe intellectual disability, suggesting a potential comorbidity between intellectual disability and seizures (1–4, 9, 12).

Of note, behavioral problem as well as autistic features were observed in most of patients. Almost all patients had a happy and/or friendly demeanor (4). Interestingly, Patient 1 was not found to have autistic behaviors and other abnormal behaviors other than exhibiting a friendly demeanor. Moreover, patient 2 exhibited previously unobserved severe behavioral problems, including cognitive impairment, hyperactivity, social anxiety, self-mutilation, impulsivity, violent behavior, and other autism-like behaviors. Therefore, patients with Skraban-Deardorff syndrome should be monitored for behavioral problems.

In this study, WES was performed in two unrelated Chinese patients with ID and DD. Eleven candidate variants in 10 genes were mapped to known phenotypes of the patients by screening using TGex software (Supplementary Table S1). The variants in the genes *LAMA1*, *PLXNA1* and *JAG2* were heterozygous. The diseases caused by these gene variants are autosomal recessive and have therefore been ruled out. The variants in the genes *MBD5*, *CDON*, *ASH1l*, *GABBR2*, *TBR1*, and *CHAMP1* were inherited from the unaffected parents. Subsequently, it is determined that they are not the causative factors for the observed phenotypes. As a result, two novel *de novo* heterozygous variants in *WDR26* were identified. c.1797delC(p.His599fs*11) is a novel frameshift variant that causes a frameshift alteration after codon 599 (Histidine) and leads to a premature termination codon that is located at codon 610. c.1414C>T(p.Gln472*) is a novel nonsense variant that also causes a premature termination. These variants were located in WD5 repeats domain of the *WDR26* and may act similarly to other LoF variants in *WDR26*, such as frameshifts, nonsense variants and splice sites result in an absence of protein production and markedly reduced mRNA levels, due to the nonsense-mediated mRNA decay (NMD) degradation (6, 8, 10). In accordance with the ACMG/AMP standards and guidelines, the c.1797delC(p.His599fs*11) and c.1414C>T(p.Gln472*) variants were pathogenic, using the PVS1, PS2 and PM2 criteria.

Limitations of our study include the limited number of cases, the lack of long-term follow-up of these patients, and the absence of functional studies of the variants. Our finding should be regarded as provisional. Investigation of a greater number of patients is expected to further refine phenotypes and determine genotypic effects and other phenotypic determinants.

The mechanism by which a haplotype deficiency of the *WDR26* gene leads to Skraban-Deardorff syndrome remains a mystery. The pattern of widespread expression of WDR26 in human tissues suggests that mutations in the *WDR26* gene may be responsible for the widespread and heterogeneous phenotype of Skraban-Deardorff syndrome (5–7). WDR26 contains WDRs, one of the most abundant protein-protein interaction structural domains, which play an important role in several biological processes (14). Among the numerous intracellular signalling pathways that WDR26 is involved in regulating, alterations in Wnt and PI3 K have been implicated in neurodevelopmental, neurodegenerative and neuropsychiatric disorders, and dysfunction in these pathways has also been associated with diseases such as cardiovascular, renal, pulmonary, allergic, bone and oral diseases (8, 9). Mutations in the *WDR26* gene also affect the assembly of the CTLH e3 complex, which is involved in the ubiquitination and proteasomal degradation of factors that maintain cellular homeostasis and are involved in cell growth, proliferation and differentiation (7, 15–20). These findings suggest that *WDR26* variants may affect neurodevelopment by interfering with multiple biological processes. A more complete understanding of Skraban-Deardorff syndrome and its mechanism of action will be achieved by further functional studies of these variants.

## Conclusion

In conclusion, this study identified two novel pathogenic variants in the *WDR26* gene in two unrelated Chinese patients. The symptoms observed in these patients were consistent with cases of Skraban-Deardorff syndrome, including varying degrees of ID, DD, speech delay, wide-foot and/or stiff-legged gait, facial dysmorphism, behavioural abnormalities, with or without seizures. Additionally, the diverse phenotypes of the patients described in the study contribute to the range of phenotypic spectrum in Skraban-Deardorff syndrome. These findings will be valuable in the genetic diagnosis of the disease and in the study of the biological mechanisms involved in the pathogenesis of Skraban-Deardorff syndrome.

## Data Availability

The datasets presented in this study can be found in online repositories. The names of the repository/repositories and accession number(s) can be found in the article/Supplementary Material.
